# Supernumerary Kidney (Triple Kidney) With Horseshoe Malformation: A Case Report

**DOI:** 10.7759/cureus.31436

**Published:** 2022-11-13

**Authors:** Shubham V Nimkar, Pallavi Yelne, Shilpa A Gaidhane, Sourya Acharya, Sunil Kumar, Nitish Batra

**Affiliations:** 1 Department of Medicine, Jawaharlal Nehru Medical College, Datta Meghe Institute of Medical Sciences, Wardha, IND

**Keywords:** congenital, anomaly, infection, horseshoe, supernumerary

## Abstract

The presence of an extra (third) kidney is an unusual congenital anomaly of the urinary system (US), having less than a hundred cases reported globally. Owing to the rare occurrence of this complex anomaly, the fused supernumerary kidney and horseshoe portion is very scarcely reported with unknown incidence. This paper presents a rare renal anomaly case of a fused supernumerary kidney with a horseshoe portion in a 41-year-old male who presented with fever, abdominal pain, and burning micturition. CT of the kidney urinary bladder showed non-rotation of the right kidney with a supernumerary malrotated horseshoe-shaped kidney and malrotated left kidney with features of acute pyelonephritis. The patient was managed with double J stenting and appropriate antibiotics till discharge.

## Introduction

An additional reniform kidney with its specific diverse vasculature, collection system, and encapsulated parenchyma is known as a supernumerary kidney (SK). The presence of an extra (third) kidney is an unusual congenital condition of the urinary system (US), having less than a hundred cases reported globally [1-3]. As the anomaly's occurrence is rare, the true incidence is unclear, but it is thought to be present in males as well as females equally [1,2].

## Case presentation

A 41-year-old male presented with right lumbar abdominal pain, burning with micturition, and high-grade fever ongoing for five days. He also had a complaint of high-grade fever for the last five days. Abdominal pain was localized to the right lumbar region. There was no blood in the urine but was turbid. Vital signs were stable on general examination with a pulse of 90/min, BP was 100/70mmHg, oxygen saturation was 95% on room air, and respiratory rate was 18/min. Abdominal examination was soft with tenderness present in the right lumbar region. No organomegaly, guarding, or rigidity was present. The rest of the systemic examination was within normal limits. The provisional diagnosis was considered to be a case of complicated urinary tract infection. 

On investigation, his hemoglobin was 9.7 g/dl, total leucocyte counts (TLCs) were raised: 16400/ cu mm, serum calcium was 6.8 g/dl, urea was slightly raised: 93 mg/dl with a creatinine of 2.4 mg/dl. All other investigations were normal. Urine routine and microscopy were s/o 4-5 pus cells/HPF and urine culture revealed growth of Klebsiella pneumonia.

Ultrasonography of the abdomen and pelvis suggested the presence of three kidneys with adjoined lower pole of all kidneys and malrotation of bilateral lumbar region kidneys: horseshoe kidney. Calculus of 11 * 11 mm (right side middle pole of the right kidney) was present.

CT of kidney urinary bladder

**Figure 1:**
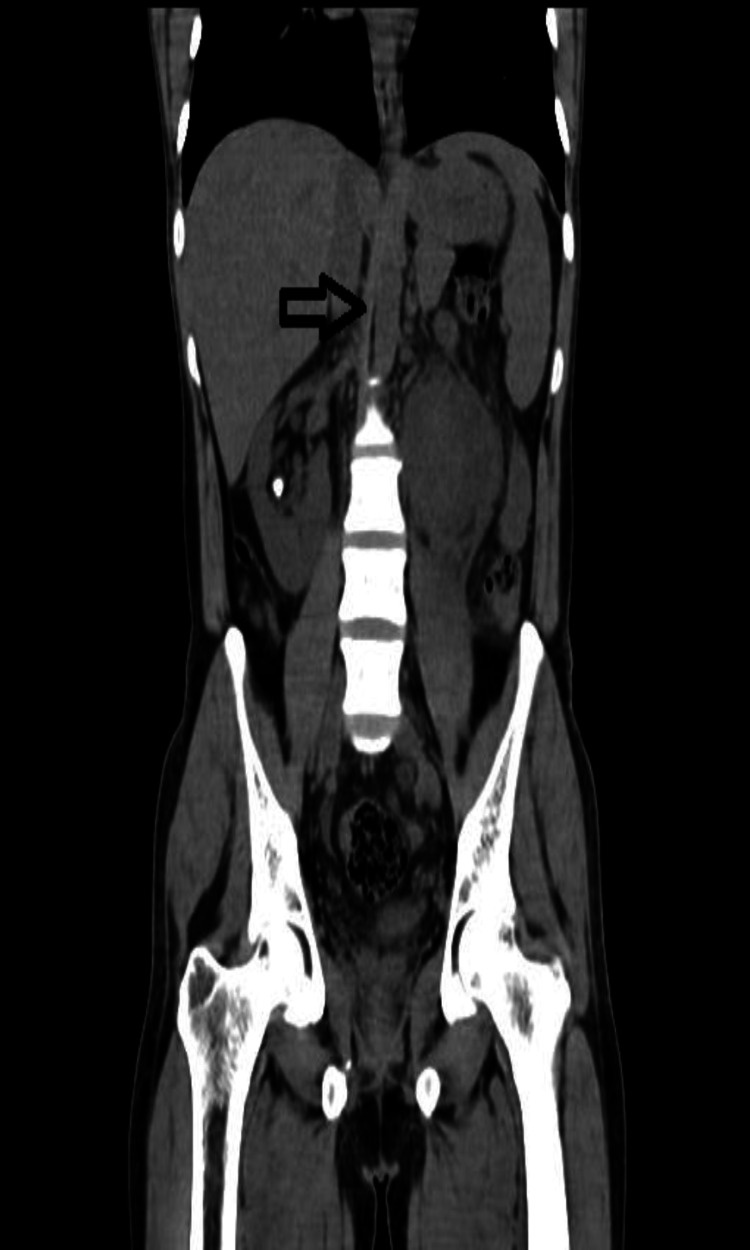
Shows the third midline kidney; the ureter of the right kidney is seen passing through its parenchyma laterally on the right

**Figure 2:**
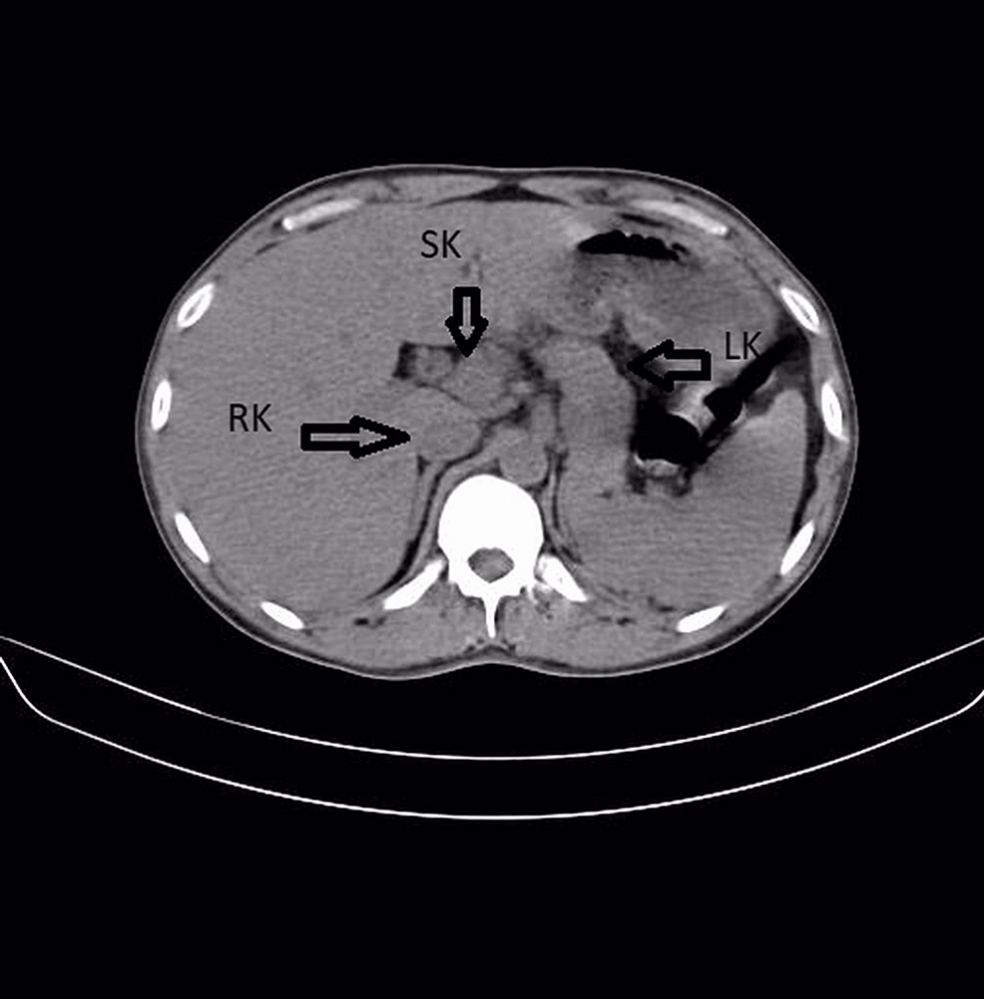
Abdominal CT scan axial image shows fusion of right kidney and supernumerary kidney anteriorly at lower poles

**Figure 3:**
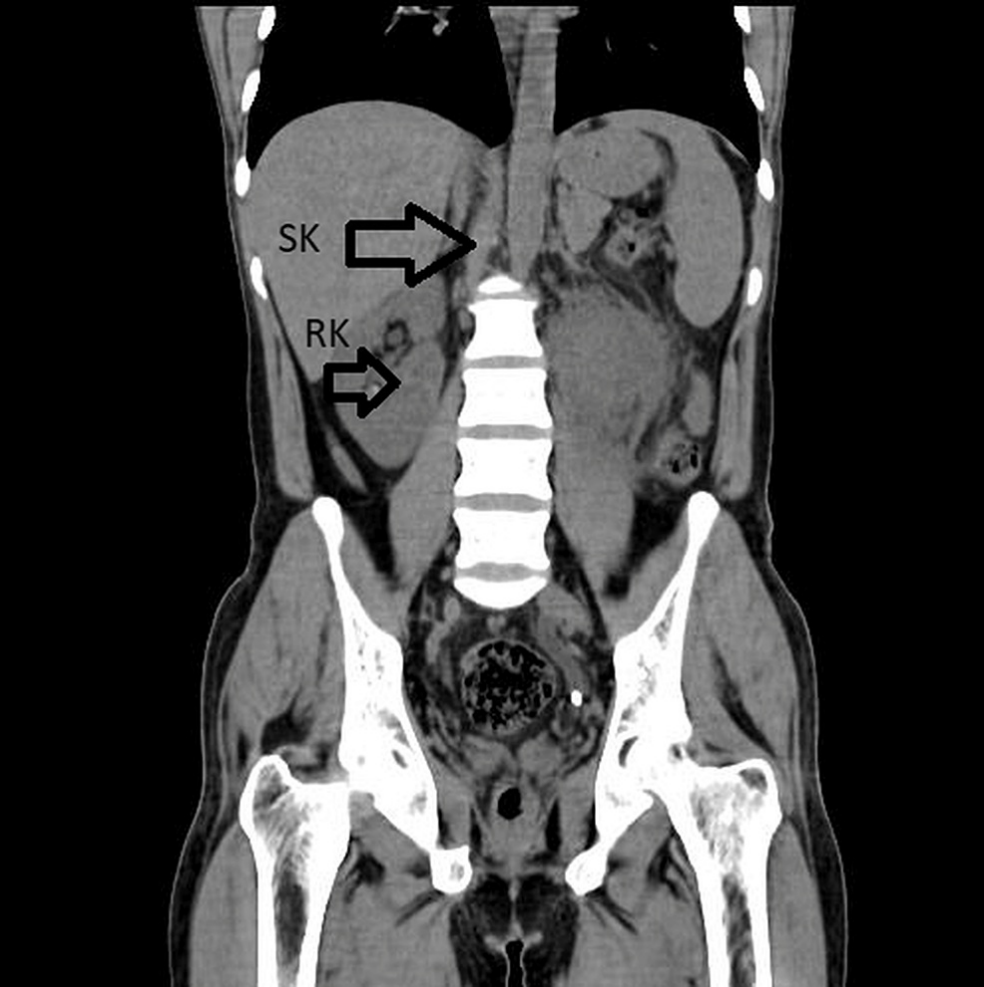
Abdominal CT scan coronal image of the abdomen showing the supernumerary kidney and the hydronephrotic right kidney

Non-rotation of right kidney with supernumerary malrotated horseshoe-shaped kidney was present. Right renal calculus was present. Malrotated left kidney with features of acute pyelonephritis with distal ureter calculus causing upstream hydroureteronephrosis was present as shown in Figure 1, 2, 3.

Management

The patient investigation report was suggestive of raised WBC count and pus cells in urinary microscopy. Ultrasonography and CT of the kidney urinary bladder were done. After considering all investigations, the patient was diagnosed with a supernumerary kidney with horseshoe malformation with bilateral renal calculi with pyelonephritis with acute kidney injury. The patient started with antibiotics like injection piperacillin and tazobactum 2.25gm IV three times a day and injection Levoflox 500mg IV once a day on alternate days and other supportive management like injection acetylcysteine 600mg IV three times a day and adequate hydration was done. Initially, creatinine was deranged and TLCs were raised. After 48 hours, he improved with the escalation of antibiotic therapy. After which left-sided double J stenting was done and the removal of the stone was planned for later once the infection cleared. Temperature, pulse, and blood pressure monitoring was done regularly. The patient was vitally stable with no active complaints and then discharged with a creatinine of 1.3. The patient was advised for follow-up after one month for double J stent removal and right renal calculi management.

## Discussion

SK may arise from irregular and abnormal division of the nephrogenic cord, which gets divided into two metanephric blastemas, which ultimately develop into two kidneys with incomplete or double ureteral bud [3-5]. Around the fifth to seventh week of gestation, when urogenital system development occurs, embryologically this anomaly used to develop. The rarity of this anomaly, its varying appearance, and the paucity of literature evidence make diagnosis and treatment difficult [6,7].

SK is an unusual urinary system congenital anomaly, with just around a hundred cases recorded in past research. It might be completely separated from the usual kidney or attached by a loose areolar tissue and it is normally smaller and less functioning as compared to normal kidneys [8]. To our knowledge, the horseshoe kidney is fairly common, found among one in 500 hundred adults, while the occurrence of the two abnormalities, i.e., SK with horseshoe kidney is extremely rare with less than five cases in the literature, globally [9,10].

SK is a rare congenital anomaly of the urogenital system. An SK is an accessory/extra or third present along with two normally located kidneys that can be presented as horseshoe kidneys.

Patient presents with complaints such as fever and abdominal pain, abdominal mass in cases and is associated with complications like hydronephrosis, pyelonephritis, pyonephrosis, renal and ureteric calculi, carcinoma, papillary cystadenoma, and Wilm’s tumor [11]. Ventricular septal defect can also present as a cardiac anomaly in congenital anomalies of the kidney and urinary tract syndrome [12]. Our patient had a supernumerary kidney with a horseshoe kidney with renal calculi, pyelonephritis, and acute kidney injury.

Diagnostic modalities employed in evaluation are intravenous pyelography, ultrasound, nuclear scintigraphy, CT, and MRI. Here we used only non-contrast CT kidney urinary bladder as the patient’s creatinine was raised up to 2.4. So contrast-enhanced CT of the kidney urinary bladder was avoided to prevent further kidney damage.

Management option depends on symptoms and complications in such a patient. Surgical interventions are performed on symptomatic cases with complications. A correct diagnosis is required for effective and successful treatment, which should eventually include uretro-nephrectomy. This drastic treatment is only recommended if acute kidney injury is there, and asymptomatic patients are closely monitored for early identification of any disease or problem inside the kidney. To summarise, there are no universally accepted treatment suggestions for the most successful therapy, save that each therapeutic option should be adapted to the particular circumstances.

## Conclusions

SK with horseshoe anomaly of the kidney even though rare, can be a cause of acute pyelonephritis. Diagnostics modalities employed in evaluation are intravenous pyelography, ultrasound, nuclear scintigraphy, CT, and MRI. Management options depend upon clinical features and complications present. Surgical interventions such as uretero-nephrectomy and double J stenting can be performed.

The embryological basis and clinical significance of this congenital anomaly are very important for the radiologist and the surgeon to know the anatomical variations in the blood supply of the horseshoe kidney as the surgery could be complicated in the presence of anomalous blood supply.

## References

[1] Suresh J, Gnanasekaran N, Dev B (2011). Fused supernumerary kidney. Radiol Case Rep.

[2] Janda GM, Nepple KG, Cooper CS, Austin JC (2009). Supernumerary kidney in a child with OEIS complex. Urology.

[3] Oto A, Kerimoğlu U, Eskiçorapçi S, Hazirolan T, Tekgül S (2002). Bilateral supernumerary kidney: imaging findings. JBR-BTR.

[4] Tada Y, Kokado Y, Hashinaka Y, Kadowaki T, Takasugi Y, Shin T, Tsukaguchi I (1981). Free supernumerary kidney: a case report and review. J Urol.

[5] N'Guessan G, Stephens FD (1983). Supernumerary kidney. J Urol.

[6] Mutlubas F, Mir S, Sevinc E, Türker Ö (2007). Unilateral double kidney with contralateral supernumerary kidney which found incidentally. Ege Tıp Dergisi / Ege Journal of Medicine.

[7] Bernik TR, Ravnic DJ, Bernik SF, Wallack MK (2001). Ectopic supernumerary kidney, a cause of para-aortic mass: case report and review. Am Surg.

[8] Ramanathan S, Kumar D, Khanna M, Al Heidous M, Sheikh A, Virmani V, Palaniappan Y (2016). Multi-modality imaging review of congenital abnormalities of kidney and upper urinary tract. World J Radiol.

[9] Mustafa M (2012). Bilateral supernumerary kidneys in conjunction with horseshoe anomaly. Saudi J Kidney Dis Transpl.

[10] Fathollahi A (2014). Supernumerary kidney with a horseshoe component. Urol Case Rep.

[11] Sagar VV, Acharya S, Kumar S, Bhawane A, Shukla S, Akhil CV, Gowda KB (2022). Right sided supernumerary kidney with pyelonephritis: a rare presentation. Journal of Research in Medical and Dental Science.

[12] Sagar VV, Acharya S, Gomase S, Singh RK, Shukla S, Kumar S, Akhil CV (2022). Ventricular septal defect (VSD) as an extra renal manifestation in congenital anomalies of kidney and urinary tract (CAKUT) syndrome: a rare case report. Medical Science.

